# Transient entanglement in minimal open XXZ spin chains: a toy-model analogy for microtubule-inspired quantum biology

**DOI:** 10.3389/fpsyt.2026.1855963

**Published:** 2026-08-11

**Authors:** Ngo Cheung

**Affiliations:** Independent Researcher, Hong Kong, Hong Kong SAR, China

**Keywords:** glutamatergic plasticity, Lindblad dynamics, microtubule stabilization, microtubule analogy, open quantum systems, preclinical falsification, quantum biology, toy model

## Abstract

Quantum approaches to consciousness remain controversial, partly because any putative quantum state in the brain is expected to decohere rapidly in a warm, hydrated, and noisy environment. The present manuscript does not attempt to solve that biological problem directly. Instead, it treats microtubule-related interpretation as a deliberately hypothetical analogy and asks a narrower Level-A question: under what generic open-system conditions can transient pairwise entanglement be enlarged in small noisy XXZ spin chains? In a minimal open XXZ spin-chain model evolved with the Lindblad master equation, stronger nearest-neighbor coupling consistently increased peak transient pairwise entanglement across several topologies and bath conditions. Among the tested environments, the memory-envelope condition gave the longest purity half-life, the collective condition increased late-time purity, and the combined bath is best described as a balanced compromise among selected diagnostics under the chosen parameters rather than as an optimized trade-off. The model is not a physical model of microtubules: it omits full 13-protofilament geometry, dynamic instability, microtubule-associated proteins, hydration structure, ionic screening, and realistic tubulin-scale calibration. On a strictly hypothetical Level-C reading, the two abstract model levers, stronger effective coupling and lower per-site dissipation, may be compared with broad classes of biological perturbation, such as glutamatergic plasticity and cytoskeletal stabilization, but not with named regimens or compounds as clinical routes to enhancement. No dosing schedule, clinical regimen, or cognitive-enhancement recommendation follows from the simulation. The proper experimental sequence is *in vitro* first, then animal work only if warranted, and human pilot work only after convergent preclinical support and safety review. Testable preclinical predictions include altered tryptophan-network fluorescence lifetimes, exciton migration signatures, superradiance-related optical behavior, and anesthetic sensitivity in controlled preparations. Negative *in vitro* optical or biophysical results would substantially weaken the biological relevance of the analogy. The manuscript should therefore be read as a reproducible toy-model study with a speculative microtubule analogy, not as evidence that pharmacological modulation can enhance cognition through quantum microtubule effects.

## Graphical Abstract

**Figure:**
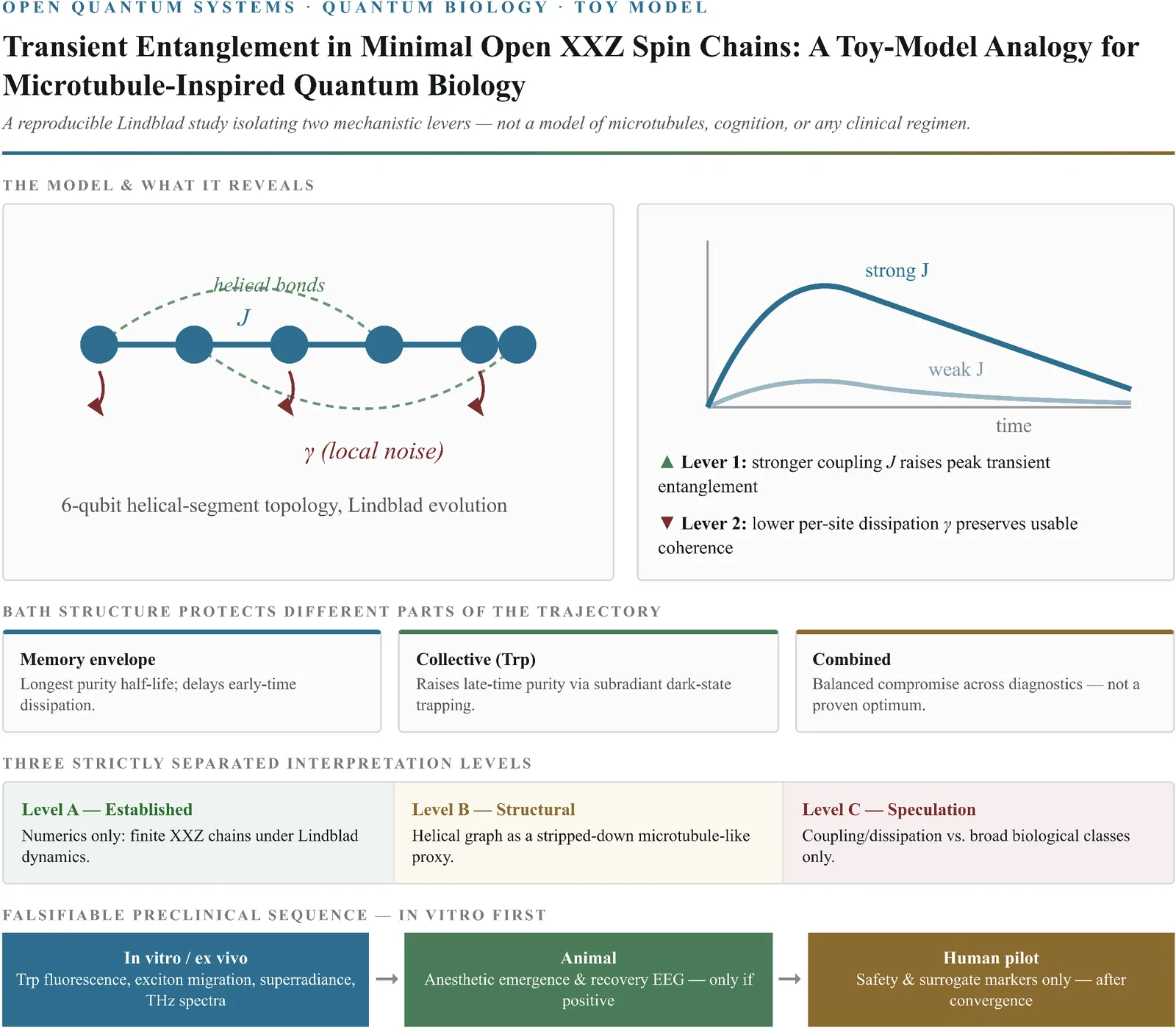

## Introduction

The hard problem of consciousness remains one of the most difficult questions in neuroscience and philosophy (1). Standard accounts describe brain function in classical terms, as electrochemical signaling across synapses, ion channels, and large-scale neural networks. Those accounts explain much, but they do not settle why physical processing should be accompanied by subjective experience or how conscious contents appear unified rather than fragmented into parallel computations. This unresolved tension has helped sustain a minority but durable line of inquiry in which quantum processes are proposed to contribute to consciousness, especially through neuronal microtubules (2).

That framing should not be overstated. The present work does not address subjective experience directly, nor does it test a theory of consciousness. It uses the consciousness literature only as historical motivation for asking a more limited open-quantum-systems question. No clinical, pharmacological, or cognitive-enhancement conclusion is drawn from that motivation.

In the orchestrated objective reduction framework, tubulin dimers inside microtubules are treated as possible sites of coherent superposition shaped by synaptic input (2). Those superpositions are then proposed to undergo objective reduction events tied to spacetime geometry, with each reduction corresponding to a discrete conscious event. The strongest objection to this idea has always been decoherence. The brain is a thermal, hydrated, fluctuating system, and such environments are usually expected to destroy quantum coherence on timescales far shorter than those relevant to neural firing or perception. Tegmark (3) gave that objection its most influential quantitative form, arguing that both ordinary neural activity and any microtubule-based quantum states would decohere too quickly to matter for cognition. Later discussions have challenged aspects of that estimate, but they have not removed the need for stringent physical modeling (4).

That criticism has not ended the debate. More recent work has kept the question open by showing that ordered biological structures can display collective optical behavior that is not easily dismissed. Reports of ultraviolet superradiance and long-range energy migration in microtubule tryptophan networks at room temperature suggest that microtubules may support collective excitonic effects under biologically relevant conditions (5, 6). Additional work indicates that volatile anesthetics interact directly with microtubule lattices, and that pharmacological stabilization of those lattices can delay anesthetic-induced loss of consciousness in rats (7). On the theoretical side, Lindblad-based modeling of tryptophan chromophore networks has identified superradiant and subradiant sectors with different consequences for information flow and correlation retention (8). A recent review has also argued that a quantum microtubule substrate remains consistent with emerging evidence and may speak to persistent issues such as perceptual binding and epiphenomenalism (9).

A separate, more field-theoretic literature has proposed biofield or field-like frameworks for neural communication (10). That line of work is mentioned here only to situate the manuscript among broader speculative attempts to move beyond purely local synaptic descriptions. The present model is distinct: it uses discrete spin-chain correlations and standard open-system dynamics rather than a continuous biofield formalism.

None of this settles the matter. The evidence does not overturn the decoherence objection, but it does suggest that biologically ordered networks can reshape how a system couples to its environment. That possibility warrants models that are simple enough to analyze but rich enough to include the main ingredients usually discussed separately, namely geometry, collective decay, memory effects, and correlated baths.

The present work adopts that narrower goal. It uses a deliberately minimal open XXZ spin-chain model implemented in QuTiP to ask how helical-style connectivity, collective decay channels, a phenomenological memory envelope, and correlated dephasing shape transient pairwise entanglement and coherence lifetime. It is a toy model. It is not a calibrated model of tubulin, microtubules, neurons, cognition, or consciousness.

The point of the simplification is practical. Real microtubules contain 13 protofilaments arranged in a helical lattice, include extended aromatic networks, and interact with structured environments that may involve ordered water and collective bath effects. A fully atomistic treatment of such systems over relevant timescales is not yet tractable. A small XXZ chain, by contrast, makes it possible to vary coupling strength, system size, topology, and bath structure in a controlled way while keeping the computations reproducible.

The interpretation used here is layered. Level-A refers only to the numerics themselves: finite XXZ chains evolving under the specified Lindblad dynamics. Level-B refers to structural analogy, such as treating the helical_segment topology as a stripped-down proxy for a microtubule-like arrangement. Level-C refers to translational speculation, including possible biological or pharmacological levers that might, in principle, map onto the two dominant parameters isolated by the model. Only Level-A is actually established by the simulations. The rest is explicit conjecture.

Three questions are therefore kept separate throughout the manuscript: first, whether small open quantum systems can show enlarged transient entanglement under favorable coupling and dissipation; second, whether real microtubules can support functionally relevant quantum correlations; and third, whether any intervention can modulate those correlations in a way that affects cognition. The simulations speak only to the first question. The second and third remain open empirical questions.

The central aim of the manuscript is therefore modest. It is to identify, within a tractable open-quantum-systems setting, which mechanistic levers most strongly favor larger transient entanglement windows. The simulations point to two such levers: stronger effective nearest-neighbor coupling and lower per-site dissipation. The remainder of the manuscript develops those findings and then considers, cautiously and hypothetically, whether broad biological perturbation classes, rather than named regimens as proposed interventions, could be used as preclinical probes of those parameters.

## Computational foundation: minimal open XXZ spin-chain models

To study the joint effects of coupling, lattice geometry, and environmental structure, a minimal open-quantum-systems pipeline was built in QuTiP (11–17). The system was modeled as an N-qubit XXZ spin chain evolving under the Lindblad master equation. The Hamiltonian contained on-site z-field terms together with nearest-neighbor XXZ couplings in the x, y, and z channels. Static Gaussian disorder was included in the on-site fields with sigma_omega = 0.15, and optional Gaussian disorder was also applied to the couplings with sigma_J = 0.15. The default anisotropy was Delta_zz = 0.2. Base coupling strength J was swept across four values, 0.5, 1.5, 4.0, and 8.0, in units where the reduced Planck constant was set to one.

Four topologies were tested by means of an explicit bond-list generator. For N = 6, the linear chain contained five nearest-neighbor bonds. The ring topology added periodic boundary conditions and therefore had six bonds. The ladder topology, defined only for even N, used rail and rung connections and yielded seven bonds. The helical_segment topology was designed as a minimal proxy for microtubule-like geometry. It combined a lateral ring with Fibonacci-step helical pathways and produced nine bonds for N = 6. These topologies increased connectivity in steps while keeping the Hilbert space manageable.

The helical_segment topology should not be read as a realistic microtubule. It captures only the abstract idea that lateral and oblique neighbor relationships may coexist in an ordered lattice. It omits dynamic instability, 13-protofilament cylindrical geometry, post-translational tubulin modifications, microtubule-associated proteins, ionic screening, hydration layers, motor traffic, lattice defects, and the many-body aromatic structure of actual tubulin polymers (18–26). For this reason, the helical_segment graph is described here as microtubule-inspired only in a structural and heuristic sense, not as a biological microtubule model.

Five bath variants were defined. The local Markovian bath served as the baseline. It applied independent amplitude-damping operators at each site with gamma_amp = 0.12 and pure-dephasing operators with gamma_dep = 0.25, using thermal occupation n_th = 0.5. The Trp_collective bath added collective radiative structure with gamma_rad = 0.08, as well as bonded-pair superradiant and subradiant lowering channels with gamma_super = 0.12 and gamma_sub = 0.02. The correlated dephasing bath introduced shared-bath sigma_z terms on bonded pairs with strength 0.3. The memory-envelope variant multiplied each collapse operator by a time-dependent envelope equal to the square root of 1 minus eta times exp(-t/tau_mem), with eta = 0.6 and tau_mem = 2.0. The combined bath stacked all four layers. In all cases, the collapse operators were embedded into the full 2^N dimensional Hilbert space by tensor products.

The term non-Markovian is used cautiously in this manuscript. The implementation is a phenomenological time-dependent Lindblad model intended to mimic delayed dissipation. It is not a full non-Markovian master equation in the sense of hierarchical equations of motion, pseudomode methods, process-tensor reconstruction, or rigorous backflow-based measures of non-Markovianity (27–30).

Each simulation began from the product coherent state |+> tensor N and evolved under QuTiP’s mesolve integrator over the interval from t = 0 to t = 12 with 300 time points. The primary QuTiP implementation used the QuTiP 5 series, with selected compatibility checks against the earlier QuTiP 2/4-style operator construction. Default mesolve tolerances were used unless otherwise specified. Selected convergence checks repeated representative runs with a denser time grid; purity half-life values changed by less than 0.3% in those checks. Disorder realizations used deterministic seeds so that each parameter set could be regenerated exactly.

The observables extracted during the run were global purity, written as Tr(rho squared), nearest-neighbor concurrence C(rho_01), negativity, quantum mutual information, the l1-norm of coherence, and the von Neumann entropy of the first qubit. Purity half-life was defined as the first time point at which purity fell below 0.5. For concurrence, the two-site reduced density matrix rho_01 was obtained by tracing out all qubits except sites 0 and 1, and Wootters’ two-qubit formula was then applied (31). Negativity was calculated from the partial transpose of the same two-site reduced state, with the bipartition taken between the two retained qubits (32, 33). Quantum mutual information was treated as a measure of total correlation, not entanglement alone. The l1-norm of coherence was treated as basis-dependent off-diagonal weight in the computational basis (34). These quantities were not treated as interchangeable with classical synchronization.

Disorder averaging used eight independent realizations. A classical Kuramoto comparator was run in parallel with an effective coupling K approximately equal to 2J (35, 36). To keep memory demands low on standard hardware, density matrices were discarded immediately after the observables were extracted.

The numerical record was cross-checked against four companion preprints that document related parameter sweeps: coupling-dependent transient entanglement, microtubule-coupling analogy, proliferation versus stabilization, and topology/bath comparisons in microtubule-inspired spin-chain models (37–40). These records are used here as computational provenance rather than as independent biological validation. The reproducibility approach follows general principles for transparent computational research and FAIR data handling (41–44).

All claims in the next section remain Level-A claims. They concern only the behavior of these finite XXZ chains under the stated Lindblad dynamics. No biological mapping is treated as established at this stage.

## Core model insights and mechanistic levers

Across the 80 primary configurations tested, defined as four topologies times five baths times four J values at N = 6, increasing nearest-neighbor coupling produced a consistent rise in peak nearest-neighbor concurrence. In the linear chain under local Markovian noise, peak concurrence rose from 0.1103 at J = 0.5 to 0.4223 at J = 8.0. The same directional pattern held in the helical_segment topology and under the combined bath, although the absolute values were lower there. Purity half-life generally shortened somewhat as J increased, which is consistent with faster coherent mixing driving the system more quickly toward the steady state set by the bath. Late-time purity itself was determined by bath structure and did not change with J.

A condensed topology comparison is shown in **Table 1**. It illustrates the main Level-A finding: stronger J amplifies transient pairwise entanglement, but extra bonds do not automatically protect coherence under local noise.

**Table 1:** Condensed topology comparison under local Markovian bath, N = 6.

Topology	Bonds	J = 0.5 peak concurrence	J = 4.0 peak concurrence	J = 8.0 peak concurrence	Final purity	Half-life range
Chain	5	0.1103	0.3988	0.4223	0.0522	0.36 - 0.28
Ring	6	0.0751	0.2415	0.2597	0.0522	0.36 - 0.28
Ladder	7	0.0570	0.1647	0.1748	0.0522	0.36 - 0.28
Helical segment	9	0.0617	0.1862	0.2025	0.0522	0.36 - 0.28

Topology mattered, but not in the most intuitive way. Under purely local Markovian noise, the linear chain outperformed the ring, ladder, and helical_segment topologies even though it had the fewest bonds. The extra connectivity in the other topologies created more pathways for coherent spreading, but it also exposed the system to more independent local decay channels. In these small systems, that additive-channel burden outweighed the gain from extra connectivity. The same pattern showed up when the system was scaled from N = 4 to N = 6. Purity half-life became shorter in every tested setting, which again fits the idea that each added qubit contributes its own dissipative operators when per-site rates are fixed.

Bath structure introduced a clearer form of protection. The memory-envelope condition produced the longest coherence windows, with purity half-life reaching 0.68 at low J and remaining 0.56 at high J. The time-dependent envelope suppressed early dissipation and preserved a protected interval of coherent evolution. The Trp_collective bath raised late-time purity to roughly five times the local Markovian floor by trapping population in subradiant dark states, but it also shortened half-life because bright-sector decay channels accelerated early losses. The combined bath brought these effects together, but it did not dominate every diagnostic. In the helical-segment case at J = 4.0, the memory-envelope condition gave higher peak concurrence, higher peak negativity, higher peak mutual information, and a longer purity half-life than the combined bath, while the Trp_collective condition gave slightly higher final purity. Because no scalar trade-off index is defined here, the combined bath is described only as a balanced compromise among the selected diagnostics under the chosen parameters.

The ablation pattern is summarized in **Table 2**. This table separates local noise, collective radiative structure, correlated dephasing, the memory envelope, and their combined effect at J = 4.0 on the helical_segment topology.

**Table 2:** Bath-component ablation on helical_segment topology, N = 6, J = 4.0.

Bath model	Main added component	Peak concurrence	Peak negativity	Peak mutual information	Final purity	Purity half-life
Local Markovian	Independent local damping and dephasing	0.1862	0.0931	0.3660	0.0522	0.28
Trp_collective	Bonded-pair superradiant and subradiant channels	0.1717	0.0857	0.3337	0.2552	0.20
Correlated dephasing	Shared bonded-pair phase noise	0.1692	0.0846	0.3296	0.0522	0.20
Memory envelope	Delayed early-time dissipation	0.2054	0.1027	0.4890	0.0500	0.56
Combined	All layers stacked	0.1918	0.0959	0.3879	0.2543	0.32

The bath comparisons are consistent with general open-systems work showing that environmental structure can sometimes support coherence or transport rather than simply destroying it (45, 46). Even so, the result remains generic open-system physics. It is not evidence that biological microtubules actually occupy the simulated regime.

A classical Kuramoto comparator showed a qualitatively similar dependence on interaction strength. Final synchronization order increased with K, in parallel with the increase in quantum concurrence as J rose (35, 36). That similarity suggests that much of the coupling dependence captured by the model reflects a general competition between interaction and noise rather than something exclusively quantum.

The quantum-specific content of the simulation is therefore narrow. Concurrence and negativity show that the simulated density matrices can contain non-separable two-qubit correlations. However, the broader trend that stronger coupling improves transient order is shared with classical synchronization models. For that reason, the Kuramoto comparator weakens any claim that coupling-dependent improvement is uniquely quantum or uniquely microtubule-relevant. This point is central to the interpretation: the coupling and dissipation result, by itself, cannot be used as evidence for a specifically quantum-biological or cognitive mechanism.

Several falsification scans sharpened the picture. Increasing gamma_dep to 5.0 and above produced complete entanglement sudden death. Positive Delta_zz on the ring suppressed concurrence by frustrating the exchange dynamics. Static disorder up to sigma = 1.0 increased run-to-run variability but did not reliably protect coherence. Initial state also mattered. Product coherent states generated entanglement, whereas a classical all-up state did not. Disorder-averaged runs across eight realizations preserved the same basic topology ranking.

A condensed robustness and falsification summary is shown in **Table 3**. The main conclusion is that early-time concurrence peaks are reproducible in the toy model, but the exact values are sensitive to bath structure, initial state, dephasing rate, and topology.

**Table 3:** Condensed robustness and falsification summary.

Test domain	Representative result	Interpretation
System size	Chain local Markovian half-life fell from 0.46 at N = 4 to 0.30 at N = 6	Larger local-noise systems decohere faster
Proliferation	In related N = 4 to 10 sweeps, half-life scaled approximately as N raised to -1.04	Added sites are liabilities under strictly local noise
Dephasing	Peak concurrence fell to zero at gamma_dep = 5.0 and 10.0	Entanglement sudden death occurs under strong dephasing
Initial state	All-up state yielded zero concurrence; embedded Bell state reached 1.0000	Results depend strongly on preparation
Disorder	Strong disorder increased variance more than protection	Disorder can sample favorable cases but is not a reliable shield
Kuramoto comparator	Classical synchronization also improved with stronger coupling	Coupling dependence is not uniquely quantum
Combined bath	Collective channels raised final purity; memory envelope extended half-life	Different bath layers protect different aspects of the trajectory; the combined bath is a balanced compromise under the chosen parameters, not a defined optimum

Taken together, the simulations isolate two dominant levers. First, stronger effective nearest-neighbor coupling helps correlations spread before local dissipation erases them. Second, reduced per-site dissipative burden is needed if that increased connectivity is to remain useful rather than self-defeating. Memory-envelope effects and collective subradiant channels act on different parts of the Liouvillian and therefore provide additive rather than interchangeable protection. These two levers, stronger coupling and lower dissipation, are the conceptual bridge to the speculative section that follows.

## Hypothetical microtubule analogy: from spin-chain levers to preclinical questions

**Figure 1:**
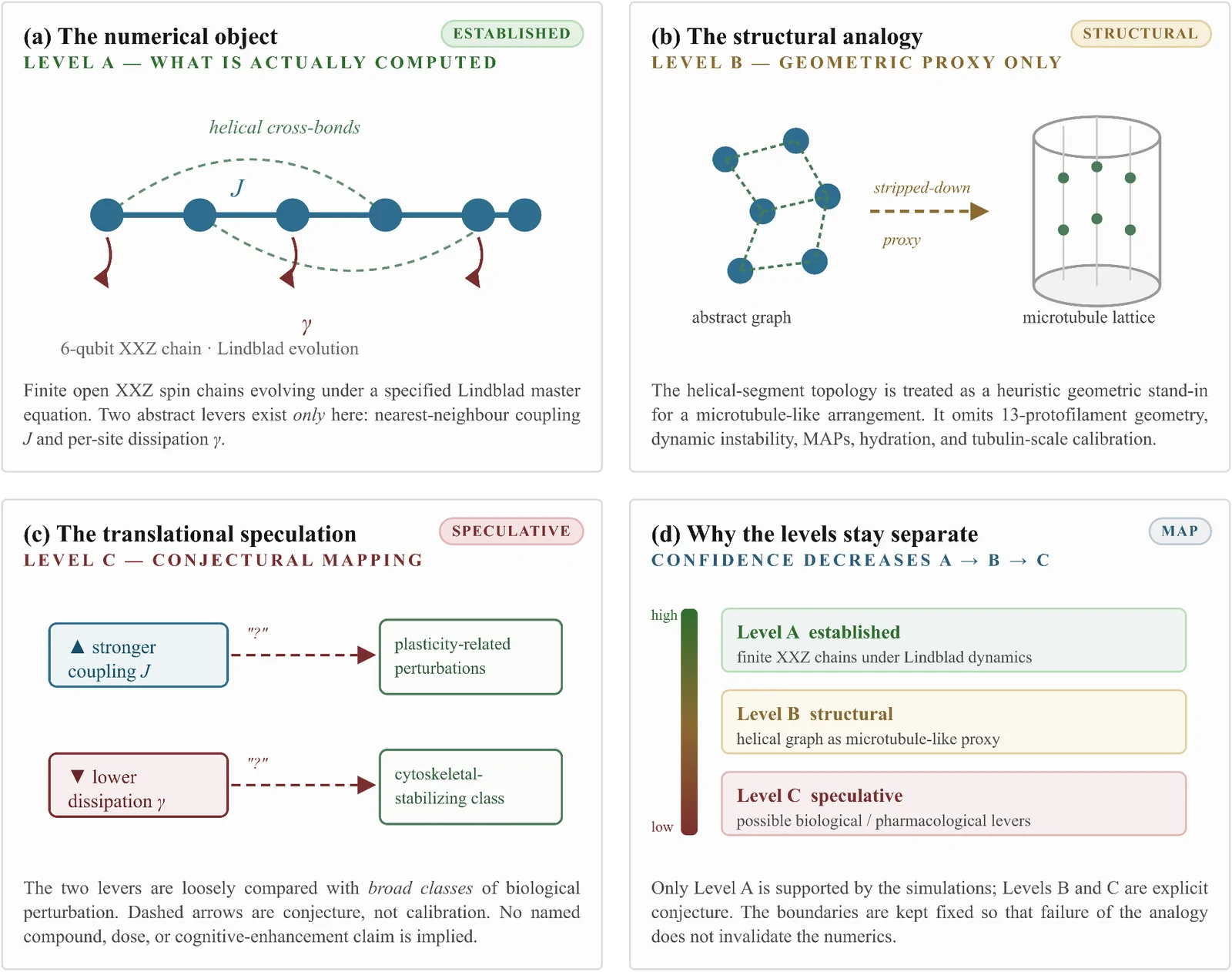
Overall conceptual overview illustrating the Level-A, Level-B, and Level-C separation. **(A)** Level A, the numerical object: a six-qubit XXZ spin chain evolved under a Lindblad master equation, in which the only well-defined quantities are the coupling lever J (blue bonds) and the per-site dissipation lever γ (red channels); dashed green lines denote helical cross-bonds. **(B)** Level B, the structural analogy: the helical-segment graph is used purely as a stripped-down geometric proxy (dashed brown arrow) for a microtubule-like lattice, with no biophysical calibration. **(C)** Level C, the translational speculation: the two levers are tentatively compared (dashed arrows, marked “?”) with broad biological classes — plasticity-related and cytoskeletal-stabilizing perturbations — without naming compounds, doses, or clinical routes. **(D)** A confidence map showing that support decreases from A to C; only Level A is established, while Levels B and C are explicit conjecture. This schematic illustrates the separation of claims only and shows no simulation results; no in vivo validation, mechanism, or cognitive-enhancement claim is made.

The move from the model to biology is hypothetical from the start (**Figure 1**). The simulations identify two abstract parameters that matter most, effective nearest-neighbor coupling and per-site dissipation. The biological question is whether any measurable feature of real tubulin or microtubule preparations behaves in a way that even loosely resembles those levers. At present, the answer is unknown. **Figure 1** separates the numerical model, broad candidate perturbation classes, and possible assay readouts. It is not a diagram of a pharmacological mechanism.

Plasticity-related perturbations form one broad class that could be examined experimentally. The source text describes the Cheung Glutamatergic Regimen, or CGR, as a proposed low-cost oral combination intended to influence glutamatergic plasticity (47, 48). In that literature, the regimen is framed as a possible way of shifting signaling balance through NMDA, AMPA, and glutamate-related pathways. Independent work on ketamine-class plasticity has linked NMDA-antagonist effects to BDNF, mTOR signaling, and synapse formation (49–53). Piracetam-related work has also been discussed in relation to neuroplasticity and mitochondrial dynamics (54). Gap-junction coupling and cytoskeletal remodeling provide additional routes by which cellular connectivity and local signaling architecture might change (55, 56).

Within the present manuscript, CGR is not treated as evidence for microtubule quantum effects. It is not treated as a route to cognitive enhancement, and it is not mapped directly onto J. If considered at all, it is only an example of a possible preclinical probe within the broader class of plasticity-related perturbations. No clinical use, self-experimentation, or cognitive-enhancement claim is implied.

A separate broad class involves cytoskeletal-stabilizing or cytoskeletal-ordering perturbations. Lithium is one example in the surrounding literature. Low-dose lithium has been discussed in both mechanistic and clinical terms as a GSK-3 beta inhibitor that can reduce tau phosphorylation and influence microtubule-related pathways (57–60). Fisetin provides a second example. It has been reported to bind beta-tubulin and affect microtubule dynamics in cellular systems, and has also been studied as a senotherapeutic compound (61, 62). In the language of this manuscript, such compounds are not proposed as interventions and are not quantitatively mapped onto gamma_amp, gamma_dep, or any other dissipation parameter. At most, they are candidate preclinical probes for asking whether controlled tubulin or microtubule preparations show reproducible optical or biophysical changes.

This analogy is qualitative, not quantitative. There is no calibration linking CGR, lithium, fisetin, or any other compound to J, gamma_amp, gamma_dep, concurrence, negativity, purity half-life, or any other model parameter. The present manuscript therefore cannot predict doses, schedules, clinical outcomes, cognitive enhancement, or consciousness-related effects.

**Figure 2:**
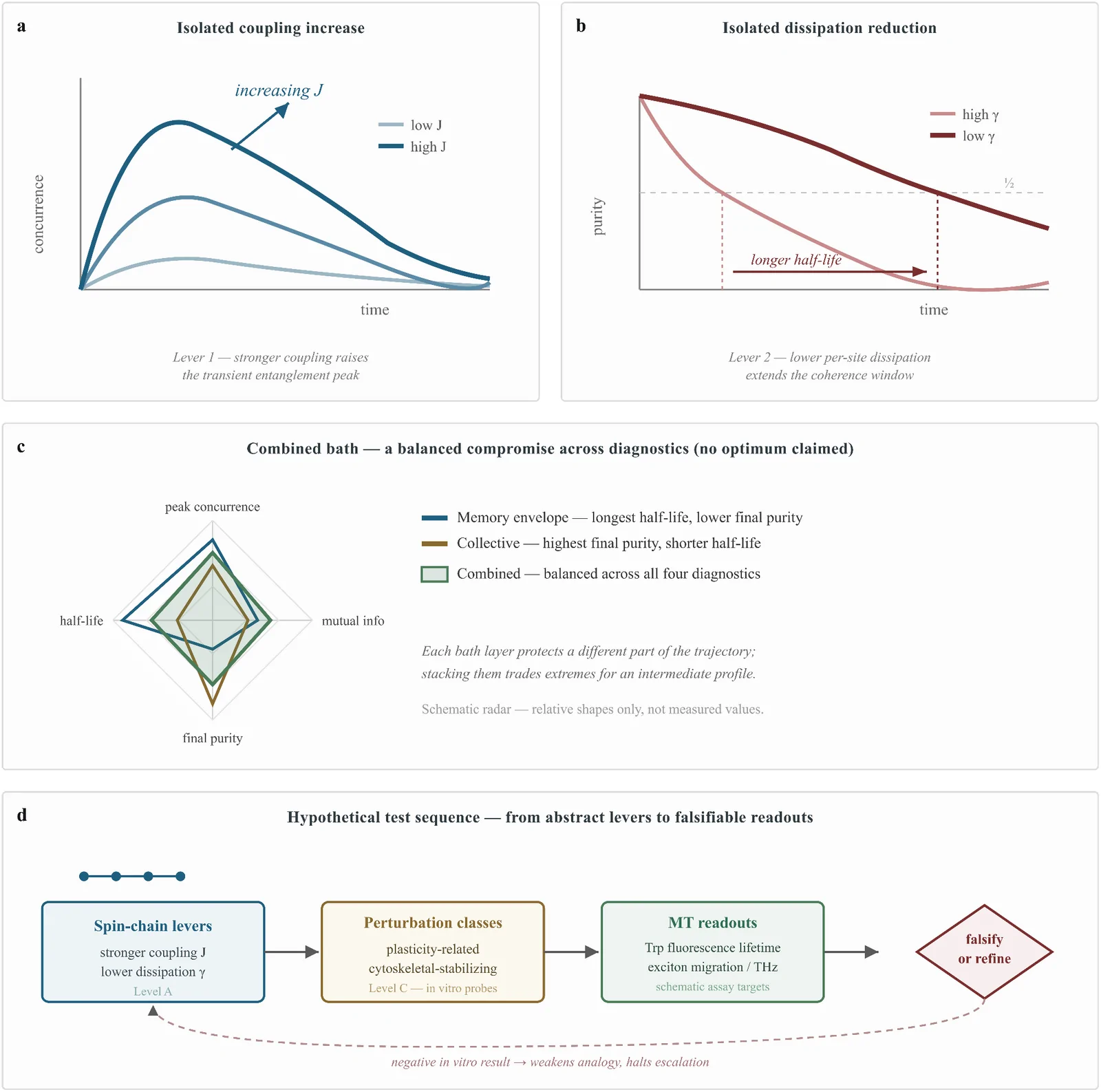
Conceptual model diagnostics and validation pathway. **(A)** Isolated coupling increase: schematic concurrence trajectories illustrating how stronger nearest-neighbor coupling J is expected to raise the transient entanglement peak. **(B)** Isolated dissipation reduction: schematic purity trajectories illustrating how lower per-site dissipation γ is expected to extend the purity half-life. **(C)** Combined bath represented as a four-axis radar (peak concurrence, mutual information, final purity, purity half-life): the memory-envelope and collective conditions occupy opposite extremes, while the combined condition is interpreted only as a balanced compromise under the chosen parameters; no trade-off optimum is claimed. **(D)** Hypothetical test sequence linking the abstract spin-chain levers to candidate in vitro perturbation classes, microtubule optical or biophysical readouts, and a falsification gate, with a feedback loop indicating that negative in vitro results halt escalation. All panels are conceptual schematics, not simulation outputs. Microtubule icons and assay labels denote targets only, not before-and-after evidence. All biological correspondences are Level-C speculation; no in vivo validation, cognitive-enhancement mechanism, or clinical recommendation is claimed.

The combined-bath results in the simulation are important because they argue against a simple “more connectivity is always better” interpretation (**Figure 2**). Under local Markovian noise, raising connectivity or system size alone shortened purity half-life, since each added site or bond effectively brought along additional decay channels. Only when per-site noise was buffered, through memory-like or collective subradiant structure, did higher connectivity remain tolerable or become beneficial. This should be read as a toy-model constraint on the interpretation of coupling, not as a claim that any biological or pharmacological perturbation can improve a microtubule lattice.

Again, this remains a toy-model warning rather than a biological result. It says that in one family of open spin chains, proliferation without stabilization is a liability. It does not show that real microtubules behave this way, and it does not show that any pharmacological intervention can move biological tissue into or out of such a regime.

## Three distinct questions

The manuscript is organized around three questions that should not be merged (**Table 4**).

**Table 4:** Separation of the computational, biological, and translational questions.

Level	Question	What the present manuscript supports	What remains unproven
Level-A	Can small open quantum systems exhibit enlarged transient entanglement under favorable coupling and dissipation?	Yes, within the specified XXZ toy models	Generality beyond these small chains
Level-B	Can real microtubules support functionally relevant quantum correlations?	Not established here	Requires direct tubulin and microtubule measurements
Level-C	Can interventions modulate such correlations to affect cognition?	Not established here	Requires *in vitro*, animal, and then human evidence

This separation is central. The computational work can be useful even if the biological and translational conjectures later fail. Conversely, any future biological result would need direct measurement rather than inference from the present spin-chain numerics.

## Preclinical research directions

The source text originally considered concrete regimen-style frameworks. Those details are not retained as manuscript recommendations. The more appropriate framing is preclinical and experimental: compounds or biological perturbations should first be tested for measurable effects on tubulin and microtubule observables before any cognitive or clinical endpoint is considered.

**Figure 3:**
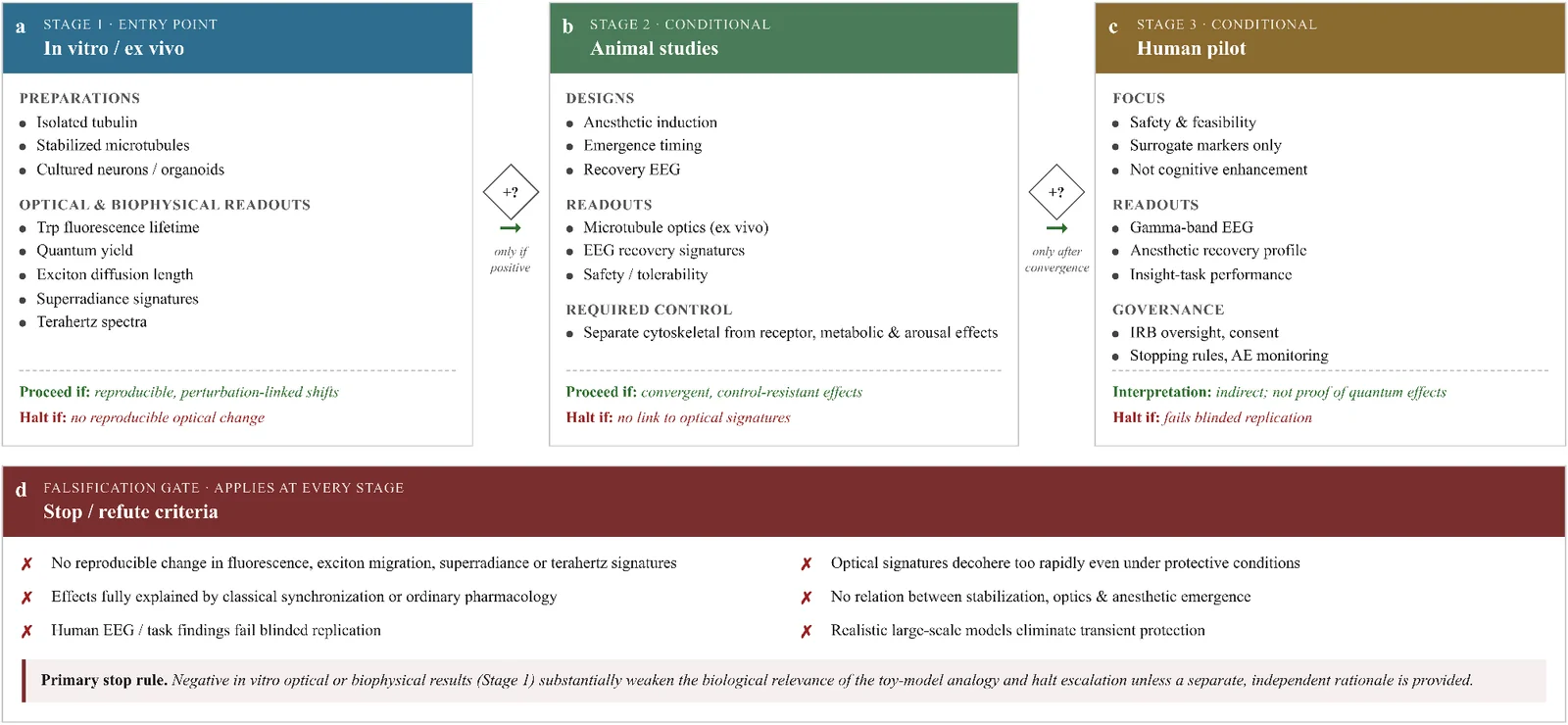
Experimental roadmap and falsification sequence. The experimental sequence runs from **(A)** in vitro and ex vivo optical and biophysical tests, to **(B)** animal anesthetic and EEG studies undertaken only if earlier results are positive, to **(C)** human safety-focused pilot work undertaken only after convergent preclinical support and safety review. Diamond gates denote conditional escalation: progression occurs only when the preceding stage yields reproducible, control-resistant effects. **(D)** Falsification criteria apply at every stage. Negative in vitro optical or biophysical results substantially weaken the biological relevance of the toy-model analogy and should stop escalation unless a separate rationale is provided. No clinical regimen, dosing schedule, or cognitive-enhancement claim follows from this roadmap; all biological correspondences are Level-C speculation only.

The first experimental step should be *in vitro* or ex vivo (**Figure 3**). Isolated tubulin, stabilized microtubule preparations, cultured neurons, or organoid-derived neuronal systems could be exposed to individual perturbations and combinations under controlled conditions. The relevant readouts would not be subjective cognition. They would be optical and biophysical measurements: tryptophan fluorescence lifetime, fluorescence quantum yield, exciton diffusion length, superradiance-like emission features, terahertz spectra, decay kinetics, and sensitivity to anesthetic exposure (5–8, 44, 63). If the model analogy has any value, perturbations that increase effective order or reduce environmental leakage should shift these readouts in reproducible directions. If such *in vitro* or ex vivo assays are negative, the biological relevance of the spin-chain analogy would be substantially weakened before any animal or human step is considered.

A second step, justified only if *in vitro* results are positive, would be animal work. The most relevant animal tests would examine anesthetic induction and emergence, EEG signatures during recovery, microtubule optical behavior in extracted tissue, and safety. Existing work showing delayed anesthetic-induced unconsciousness after microtubule stabilization provides a useful but limited precedent (7). Animal studies would need to distinguish cytoskeletal effects from ordinary receptor, metabolic, sedative, or arousal effects.

A third step, justified only after convergent preclinical evidence, would be carefully monitored human pilot work. Such work should focus on safety, feasibility, and surrogate markers rather than cognitive enhancement. Possible readouts include resting and task-related gamma-band EEG, anesthetic recovery profiles in tightly controlled contexts, and performance on insight tasks such as compound remote-associate or spatial insight paradigms (64–69). Even then, any human result would be indirect. Changes in EEG or task performance would not prove microtubule quantum effects.

## Falsifiability criteria

The hypothesis should be considered weakened or refuted if any of the following occur.

Negative *in vitro* optical or biophysical results are not a minor inconvenience in this framing; they would directly undercut the proposed biological relevance of the Level-C analogy.

- *In vitro* tubulin or microtubule preparations show no reproducible change in fluorescence lifetime, quantum yield, exciton migration, superradiance-like signatures, or terahertz spectra after stabilization or plasticity-related perturbations.
- Microtubule preparations decohere or lose optical signatures too rapidly even under conditions meant to approximate the combined-bath protection in the toy model.
- Any observed optical or EEG changes are fully explained by classical synchronization, ordinary receptor pharmacology, metabolic state, temperature, sedation, arousal, or nonspecific toxicity.
- Animal anesthetic studies show no relation between microtubule stabilization, optical signatures, and emergence dynamics.
- Human EEG or cognitive findings, if ever tested, fail to replicate under blinded conditions or show no relation to preclinical microtubule readouts.
- Larger and more realistic computational models with protofilament geometry, dynamic instability, hydration structure, and microtubule-associated proteins eliminate the transient protection seen in the toy chains.

These criteria are important because the model is not meant to protect the hypothesis from failure. It is meant to make the chain of assumptions explicit enough to test.

## Safety considerations, limitations, and ethical notes

The source text places strong emphasis on safety, and that emphasis is warranted. Even when discussed only as possible preclinical perturbations, several compounds mentioned in the broader literature have recognized risks. Dextromethorphan exposure can be increased substantially when combined with CYP2D6 inhibitors, and serotonergic toxicity is a known concern with serotonergic combinations (64–67). Lithium compounds raise concerns about renal function, thyroid function, serum-level monitoring, toxicity, and drug interactions, especially with dehydration, NSAIDs, diuretics, and other medical comorbidities (65, 68–71). Fisetin appears better tolerated in some pulsed research contexts, but long-term human data remain limited (62). Epothilone-class agents remain inappropriate for routine human use outside approved indications or formal research settings because of toxicity risks, including neuropathy and hematologic effects (7).

No part of this manuscript should be interpreted as medical advice, a treatment recommendation, or support for self-experimentation. The manuscript does not propose a clinical regimen. It proposes a hierarchy of experiments, beginning with non-human and *in vitro* tests.

The computational model has its own limitations, and these are substantial. It uses only N = 4 to 6 qubits in the primary topology and bath simulations, with related N = 4 to 10 local-noise scaling explored in companion work (37–40). The noise rates are illustrative rather than biologically measured. The helical_segment topology is an abstract graph, not a microtubule. The bath models are simplified and partly phenomenological. The memory-envelope condition is not a rigorous non-Markovian master equation. The combined bath is a stacked numerical construction, not a measured biological environment.

Real microtubules contain thousands of tubulin dimers arranged across 13 protofilaments and exhibit dynamic instability, microtubule-associated protein binding, post-translational modifications, motor-protein traffic, lattice defects, ionic screening, and hydration effects far more complex than the model allows (18–26). The additive-channel liability observed in the helical_segment topology under local noise may therefore misestimate what happens in actual tissue. The Kuramoto comparison also serves as a warning, since it shows that some coupling-dependent effects are generic consequences of interaction under noise rather than signatures of uniquely quantum biology.

The biological mapping is therefore qualitative and speculative. No quantitative conversion exists between J and synaptic density, gap-junction conductance, dipole coupling, tubulin exciton hopping, or any pharmacological effect. No quantitative conversion exists between gamma and microtubule disorder, hydration, thermal noise, anesthetic binding, or cytoskeletal stabilization.

The ethical framework is equally important. Any move beyond thought experiment would require physician supervision where human exposure is involved, written informed consent, institutional review board oversight, adverse-event monitoring, prespecified stopping rules, and a clear demand for convergent evidence across multiple methods before even small human pilots could be justified (72–74). No named compound or combination should be presented as an established intervention. In the manuscript’s own terms, it remains a Level-C hypothesis only. Independent replication, larger-N simulation work, and open reporting of negative as well as positive results are essential.

## Conclusion and future directions

The main contribution of the model is not a claim about consciousness, but the identification of two simple levers in a controlled open-quantum-systems setting. Stronger nearest-neighbor coupling increases peak transient pairwise entanglement, while lower per-site dissipation preserves usable coherence long enough for that entanglement to emerge. When these two levers are addressed together, as in the combined-bath simulations, the model yields a balanced compromise among selected diagnostics under the chosen parameters, not a proven optimum or a defined best trade-off.

The translational section does no more than ask whether those two levers might have rough biological analogs. Plasticity-related perturbations are treated as possible analogs of increased effective coupling, and cytoskeletal-stabilizing perturbations are treated as possible analogs of reduced per-site dissipation. Named compounds or regimens are not treated as validated mechanisms or proposed interventions. These are analogies, not validated mechanisms. No *in vivo* validation is claimed.

Future work could move in two directions at once. On the computational side, larger models could incorporate more explicit protofilament geometry, dynamic instability, hydration structure, tryptophan-network detail, and microtubule-associated proteins as hardware and algorithms improve. On the experimental side, the most direct starting points would be *in vitro* optical studies, terahertz or fluorescence-based measurements of microtubule excitations, and only then, if justified, animal and human studies focused on safety and surrogate markers such as gamma-band EEG or anesthetic emergence timing (5–8, 64–69).

The Kuramoto comparator keeps the conclusion deliberately conservative: stronger coupling improving transient order is not uniquely quantum and cannot by itself support a quantum-biological, cognitive, or psychopharmacological mechanism. In that sense, the value of the present manuscript is mainly heuristic. It offers a reproducible toy-model baseline, a clear statement of the underlying analogy, and a set of predictions that could in principle fail. The proposed biological interpretation remains a thought experiment only. The appropriate endpoint is preclinical falsification or support, beginning with *in vitro* optical and biophysical assays, not a psychopharmacological or cognitive-enhancement proposal. Progress in this area will depend on careful empirical work, transparent reporting, and respect for the large distance that still separates small spin-chain numerics from the biology of the living brain.

## Data Availability

The original contributions presented in the study are included in the article/supplementary material. Further inquiries can be directed to the corresponding author.
